# Supplementary material for: Coevolution-based prediction of key allosteric residues for protein function regulation
Source: eLife. 2023 Feb 17;12:e81850. doi: 10.7554/eLife.81850 (PMC9981151; doi:10.7554/eLife.81850)
Supplement: Supplementary file 1. [file elife-81850-supp1.docx]

**Supplementary File 1-Information of the allosteric proteins in the data set**

**Supplementary File 1**. Information of the allosteric proteins in the data set

| Protein name | PDB ID_AS^a^ | PDB ID_OS | Allosteric effector | Orthosteric effector | Organism | Allosteric type |
| --- | --- | --- | --- | --- | --- | --- |
| Androgen receptor (AR1) | 2YHD | 2PIO | AV6 | DHT | Homo sapiens | Inhibitor |
| Androgen receptor (AR2) | 2YLO | 2PIO | YLO | DHT | Homo sapiens | Inhibitor |
| Beta-lactamase TEM (TEM-1) | 1PZO | 1AXB | CBT | FOS | Escherichia coli | Inhibitor |
| Casein kinase II subunit alpha (CK2alpha) | 3H30 | 3H30 | RFZ | RFZ | Homo sapiens | Inhibitor |
| Cell division control protein 4 (Cdc4) | 3MKS | 1NEX | C1C | TPO | Saccharomyces cerevisiae | Inhibitor |
| Cyclin-dependent kinase 2 (CDK2) | 3PXF | 1B38 | 2AN | ATP | Homo sapiens | Inhibitor |
| Cytochrome P450 3A4 (CYP3A4) | 1W0F | 1W0F | STR | HEM | Homo sapiens | Regulator |
| Focal adhesion kinase 1 (FADK 1) | 4EBW | 2IJM | 0PF | ATP | Homo sapiens | Inhibitor |
| Glucokinase (HK4) | 1V4S | 3F9M | MRK | GLC | Homo sapiens | Activator |
| Glutamate racemase (MurI) | 2JFN | 2JFZ | UMA | DGL | Escherichia coli | Activator |
| Gtpase hras (Ha-Ras) | 4DLR | 4DLR | DTU | GNP | Homo sapiens | Regulator |
| Insulin-like growth factor 1 receptor (IGF-1R) | 3LW0 | 1JQH | CCX | ANP | Homo sapiens | Inhibitor |
| Isocitrate dehydrogenase kinase/phosphatase (AceK) | 3LCB | 3LCB | AMP | ATP | Escherichia coli | Inhibitor |
| Kinesin-like protein kif11 (TRIP-5) | 3ZCW | 3ZCW | 4A2 | ADP | Homo sapiens | Inhibitor |
| Mitogen-activated protein kinase 14 (MAPK14) | 4E6C | 3KF7 | 0O8 | L9G | Homo sapiens | Activator |
| Mitogen-activated protein kinase 8 (MAPK8) | 3O2M | 1UKI | 46A | 537 | Homo sapiens | Inhibitor |
| Supplementary File 1. Continued | |  |  |  |  |  |
| Myosin-2 heavy chain (Myosin-2) | 2JHR | 1YV3 | PBQ | ADP | Dictyostelium discoideum | Inhibitor |
| Protein RecA (RecA) | 2G88 | 2G88 | DTP | DTP | Mycolicibacterium smegmatis | Regulator |
| Protein-tyrosine phosphatase 1B (PTP-1B) | 1T49 | 1BZC | 892 | TPI | Homo sapiens | Inhibitor |
| RAC-alpha serine/threonine-protein kinase (PKB) | 3O96 | 4EKK | IQO | ANP | Homo sapiens | Inhibitor |
| RTX toxin RtxA (MARTX) | 3GCD | 3GCD | IHP | AZ0 | Vibrio cholerae | Regulator |
| Serine/threonine-protein kinase Chk1 (CHK1) | 3F9N | 2E9N | 38M | 76A | Homo sapiens | Inhibitor |
| Tyrosine protein kinase ABL1 (Bcr-Abl) | 3K5V | 3K5V | STJ | STI | Mus musculus | Inhibitor |
| Tyrosine-protein kinase ABL1 (BCR-ABL1) | 5MO4 | 5MO4 | AY7 | NIL | Homo sapiens | Inhibitor |
| Tyrosine-protein kinase ABL1 (c-Abl) | 3PYY | 3PYY | 3YY | STI | Homo sapiens | Activator |
| ^a^AS, allosteric site; OS, orthosteric site; PDB ID_AS, protein structure in the allosteric effector-bound state; PDB ID_OS, protein structure used to define OS. | | | | | | |
