# Supplementary material for: Coevolution-based prediction of key allosteric residues for protein function regulation
Source: eLife. 2023 Feb 17;12:e81850. doi: 10.7554/eLife.81850 (PMC9981151; doi:10.7554/eLife.81850)
Supplement: Supplementary file 2. [file elife-81850-supp2.docx]

**Supplementary File 2-List of the Z-scores and ranking of allosteric pockets in the data set**

**Supplementary File 2**. List of the Z-scores and ranking of allosteric pockets in the data set

| Protein name | Z-score^a^ | Rank^b^ |
| --- | --- | --- |
| Cdc4 | 2.47 | 1/11 |
| MARTX | 1.67 | 1/4 |
| Ha-Ras | 0.61 | 2/3 |
| TEM-1 | 1.99 | 1/7 |
| MurI | 2.17 | 1/7 |
| AR1 | 0.09 | 3/7 |
| IGF-1R | 2.23 | 1/8 |
| Bcr-Abl | 2.05 | 1/10 |
| PTP-1B | 1.30 | 3/12 |
| c-Abl | 1.36 | 3/11 |
| AR2 | 1.13 | 2/7 |
| FADK 1 | 2.61 | 1/8 |
| CDK2 | 3.03 | 1/15 |
| CHK1 | 0.90 | 3/9 |
| CK2alpha | 1.49 | 1/15 |
| TRIP-5 | 2.16 | 1/11 |
| RecA | 2.11 | 2/13 |
| MAPK14 | 2.39 | 1/11 |
| MAPK8 | 0.63 | 3/16 |
| PKB | 3.06 | 1/12 |
| HK4 | 2.45 | 1/10 |
| CYP3A4 | 0.11 | 8/14 |
| BCR-ABL1 | 1.49 | 2/15 |
| AceK | 2.63 | 1/18 |
| Myosin-2 | 1.80 | 2/24 |
| ^a^Z-score: the Z-score corresponding to the evolutionary coupling strength of the allosteric pocket. ^b^Rank: the ranking of allosteric pocket in all pockets except orthosteric pockets according to Z-score in KeyAlloSite. | | |
