# Supplementary material for: Coevolution-based prediction of key allosteric residues for protein function regulation
Source: eLife. 2023 Feb 17;12:e81850. doi: 10.7554/eLife.81850 (PMC9981151; doi:10.7554/eLife.81850)
Supplement: Supplementary file 3. [file elife-81850-supp3.docx]

**Supplementary File 3-KeyAlloSite prediction results of Aurora A kinase**

**Supplementary File 3**. KeyAlloSite prediction results of Aurora A kinase

| PPI | |  |  | Phosphorylation | |
| --- | --- | --- | --- | --- | --- |
| Pockets | Z-score |  | Residues | Sites | Z-score |
| cavity_1 | 1.95 |  | THR | 235 | 1.36 |
| **cavity_3** | **1.48** |  | SER | 245 | 1.23 |
| cavity_6 | 0.80 |  | SER | 249 | 1.09 |
| cavity_7 | 0.50 |  | **THR** | **287** | **1.05** |
| cavity_9 | -0.27 |  | **THR** | **288** | **0.83** |
| cavity_12 | -0.39 |  | THR | 204 | 0.82 |
| cavity_11 | -0.51 |  | SER | 361 | 0.70 |
| cavity_10 | -0.54 |  | TYR | 148 | 0.67 |
| cavity_8 | -0.56 |  | SER | 369 | 0.60 |
| cavity_4 | -1.11 |  | SER | 226 | 0.60 |
| cavity_5 | -1.34 |  | SER | 155 | 0.49 |
|  |  |  | TYR | 212 | 0.47 |
|  |  |  | TYR | 219 | 0.32 |
|  |  |  | THR | 384 | 0.17 |
|  |  |  | THR | 353 | 0.12 |
|  |  |  | THR | 347 | 0.06 |
|  |  |  | TYR | 338 | -0.26 |
|  |  |  | TYR | 334 | -0.29 |
|  |  |  | SER | 283 | -0.34 |
|  |  |  | THR | 292 | -0.44 |
|  |  |  | SER | 284 | -0.48 |
|  |  |  | SER | 266 | -1.33 |
|  |  |  | THR | 333 | -1.33 |
|  |  |  | SER | 342 | -1.91 |
|  |  |  | SER | 387 | -1.97 |
|  |  |  | SER | 123 | -2.23 |
