# Supplementary material for: Coevolution-based prediction of key allosteric residues for protein function regulation
Source: eLife. 2023 Feb 17;12:e81850. doi: 10.7554/eLife.81850 (PMC9981151; doi:10.7554/eLife.81850)
Supplement: Supplementary file 4. [file elife-81850-supp4.docx]

**Supplementary File 4-List of the predicted key allo-residues in allosteric pockets**

**Supplementary File 4**. List of the predicted key allo-residues in allosteric pockets

| Protein | Predicted key allo_residues^a^ |
| --- | --- |
| Ha-Ras | A/62,A/89,A/94 |
| Cdc4 | D/662,D/634 |
| MARTX | A/200,A/135,A/85,A/54,A/51,A/201,A/168,A/180,A/167 |
| AR1 | A/736,**A/732**,A/725 |
| AR2 | A/724,A/833,A/722,**A/832**,A/721,A/826 |
| TEM-1 | A/246,A/245,A/217,A/214,A/248,A/233,A/216 |
| MurI | A/229,A/237,A/233,A/150,A/123,A/96,A/226,A/151,A/116,A/235,A/101 |
| Bcr-Abl | A/523,A/453,A/450,A/352,A/356,A/479,A/360,**A359**,A358,A351 |
| PTP-1B | A/195,A/187,**A/282** |
| c-Abl | A/478,**A/359**,A/519,A/482,A/455,A/364,A/481 |
| IGF-1R | A/1136,A/1135,A/1167,A/1154,A/1039,A/1155,A/1045,A/1156 |
| FADK 1 | A/546,A/621,A/618,A/540,A/598,A/475,A/545,A/535,A/474,A/544,A/541,A/668,A/472,A/548 |
| CDK2 | A/147,A/51,A/154,A/125,A/148,A/158,A/157,A/35,**A/155**,A/71,A/61 |
| CHK1 | A/233,A/132,A/205 |
| CK2alpha | **A/54**,A/104,A/59,A/36,**A/110** |
| TRIP-5 | A/115,A/117,A/134,A/235,A/232,A/202,A/162,A/227,A/213,A/164,A/116,A/170 |
| RecA | A/54,A/328,A/50,A/52,A/337,A/257,A/256,A/247,A/255,A/56,A/254,A/49,A/246,A/53 |
| MAPK14 | A/200,**A/192,A/191**,A/296,A/194,A/240,A/293,A/207,A/193 |
| MAPK8 | A/197,A/255,A/257,A/196,A/185,A/251,**A/195**,A/192,**A/200** |
| PKB | A/273,A/331,A/202,A/275,A/213,A/18,A/14 |
| HK4 | A/457,A/460,A/69,A/461,A/459,A/96,A/453,A/248,A/207,A/201 |
| CYP3A4 | A/484,A/482,A/308,A/304,**A/219** |
|  |  |
|  |  |
| Supplementary File 4. Continued | |
| BCR-ABL1 | A/479,A/525,A/454,A/450,**A/359**,A/453,A/364 |
| AceK | A/59,A/56,A/291,A/256,A/414,A/412,A/372,A/294,A/371,A/299,A/114,A/104,A/374,A/361,A/298,A/384,A/120,A/100 |
| Myosin-2 | A/240,A/468,A/267,A/264,A/471,A/435,A/262,A/259,A/239,A/635,A/430,A/632,A/594,A/588,A/263,A/261,A/587,A/426,A/438 |
| ^a^Predicted key allo_residues: Chain/Residue sequence number. Among the predicted key allo-residues, the residues that have been annotated as functional residues by experimental data in the literature are marked in bold. | |
