# Supplementary material for: Coevolution-based prediction of key allosteric residues for protein function regulation
Source: eLife. 2023 Feb 17;12:e81850. doi: 10.7554/eLife.81850 (PMC9981151; doi:10.7554/eLife.81850)
Supplement: Supplementary file 5. [file elife-81850-supp5.docx]

**Supplementary File 5-** **Key allo-residues predicted by KeyAlloSite with different cutoffs**

**Supplementary File 5**. Key allo-residues predicted by KeyAlloSite with different cutoffs

| Proteins | Known key allo-residues | cutoff0.5 | cutoff0.6 | cutoff0.7 | cutoff0.8 | cutoff0.9 | cutoff1.0 |
| --- | --- | --- | --- | --- | --- | --- | --- |
| BCR-ABL1 | L359 | L359 | L359 | L359 | L359 | L359 | L359 |
| Tar | Y149, Q152 | Y149, Q152 | Y149, Q152 | Y149, Q152 | Y149, Q152 | N | N |
| PDZ3 | A347, L353 | A347, L353 | A347, L353 | A347, L353 | A347, L353 | A347 | A347 |
