# Supplementary material for: Coevolution-based prediction of key allosteric residues for protein function regulation
Source: eLife. 2023 Feb 17;12:e81850. doi: 10.7554/eLife.81850 (PMC9981151; doi:10.7554/eLife.81850)
Supplement: Supplementary file 6. [file elife-81850-supp6.docx]

**Supplementary File 6-KeyAlloSite prediction results of tyrosine-protein kinase ABL1**

**Supplementary File 6**. KeyAlloSite prediction results of tyrosine-protein kinase ABL1

| Residues^a^ | Z-score |  | Residues^a^ | Z-score |
| --- | --- | --- | --- | --- |
| **R479** | **2.35** |  | L448 | -0.43 |
| **V525** | **1.97** |  | F516 | -0.43 |
| **Y454** | **1.97** |  | E353 | -0.56 |
| **E450** | **1.85** |  | V358 | -0.56 |
| **L359** | **1.72** |  | L360 | -0.56 |
| **T453** | **1.72** |  | L447 | -0.56 |
| **T364** | **1.47** |  | A452 | -0.56 |
| C483 | 0.71 |  | Y488 | -0.56 |
| A356 | 0.58 |  | V354 | -0.68 |
| E526 | 0.58 |  | M362 | -0.68 |
| Q352 | 0.45 |  | W449 | -0.68 |
| L490 | 0.45 |  | M456 | -0.68 |
| M515 | 0.45 |  | M491 | -0.68 |
| I521 | 0.45 |  | V357 | -0.81 |
| D523 | 0.45 |  | A363 | -0.81 |
| L529 | 0.45 |  | P480 | -0.81 |
| G455 | 0.33 |  | V487 | -0.81 |
| S522 | 0.20 |  | F512 | -0.81 |
| E524 | 0.07 |  | I451 | -1.19 |
| Y361 | -0.05 |  | E481 | -1.32 |
| P484 | -0.3 |  | G482 | -1.57 |
| R351 | -0.43 |  | N355 | -1.70 |
| ^a^Residues: Among all residues in the allosteric pocket, the predicted key allo-residues are marked in bold. | | | | |
