# Supplementary material for: Coevolution-based prediction of key allosteric residues for protein function regulation
Source: eLife. 2023 Feb 17;12:e81850. doi: 10.7554/eLife.81850 (PMC9981151; doi:10.7554/eLife.81850)
Supplement: Supplementary file 7. [file elife-81850-supp7.docx]

**Supplementary File 7-The key allo-residues predicted by our method on *Candida antarctica* lipase B**

**Supplementary File 7**. The key allo-residues predicted by our method on *Candida antarctica* lipase B

| Key allo-residues^a^ | Z-score | Key allo-residues^a^ | Z-score |
| --- | --- | --- | --- |
| **P133** | 3.98 | A305 | 1.58 |
| L73 | 3.94 | G44 | 1.54 |
| **A225** | 3.04 | **A146** | 1.52 |
| **S201** | 3.04 | G60 | 1.52 |
| S150 | 3.04 | T186 | 1.46 |
| **E188** | 3.00 | R249 | 1.44 |
| **E269** | 2.72 | **T244** | 1.44 |
| **S47** | 2.51 | S153 | 1.44 |
| **N169** | 2.33 | **V37** | 1.42 |
| S250 | 2.31 | R309 | 1.36 |
| V78 | 2.31 | W113 | 1.34 |
| **M72** | 2.31 | S195 | 1.30 |
| P152 | 2.25 | L90 | 1.21 |
| S67 | 2.25 | F205 | 1.19 |
| S243 | 2.21 | L204 | 1.15 |
| L140 | 2.13 | P63 | 1.09 |
| T238 | 2.05 | Q112 | 1.01 |
| **V110** | 2.05 | **I255** | 0.99 |
| **Q191** | 1.99 | L167 | 0.99 |
| **K290** | 1.95 | **L144** | 0.97 |
| **L228** | 1.93 | L128 | 0.97 |
| **P218** | 1.88 | T88 | 0.97 |
| S184 | 1.86 | **A8** | 0.97 |
| I66 | 1.82 | Y203 | 0.95 |
| P260 | 1.80 | S161 | 0.93 |
| **D187** | 1.76 | I87 | 0.93 |
| ^a^Key allo-residues: Among the predicted key allo-residues, the residues that have been annotated as functional residues by experimental data in the literature are marked in bold. | | | |
