# Supplementary material for: Coevolution-based prediction of key allosteric residues for protein function regulation
Source: eLife. 2023 Feb 17;12:e81850. doi: 10.7554/eLife.81850 (PMC9981151; doi:10.7554/eLife.81850)
Supplement: Supplementary file 8. [file elife-81850-supp8.docx]

**Supplementary File 8-The confusion matrices of KeyAlloSite in different scenarios**

**Supplementary File 8**. The confusion matrices of KeyAlloSite in different scenarios

| **Prediction of allosteric sites** | | | |
| --- | --- | --- | --- |
| Confusion matrix | | Predict | |
|  |  | Positive | Negative |
| Real | Positive^a^ | 23 | 2 |
|  | Negative^b^ | 41 | 212 |
| **Prediction of key allo-residues in allosteric sites** | | | |
| Confusion matrix | | Predict | |
|  |  | Positive | Negative |
| Real | Positive^a^ | 5 | 0 |
|  | Negative^b^ | 13 | 76 |
| **Prediction of PTM** | | | |
| Confusion matrix | | Predict | |
|  |  | Positive | Negative |
| Real | Positive^a^ | 2 | 1 |
|  | Negative^b^ | 8 | 15 |
| **Prediction of pathogenetic mutations** | | | |
| Confusion matrix | | Predict | |
|  |  | Positive | Negative |
| Real | Positive^a^ | 11 | 21 |
|  | Negative^b^ | 40 | 174 |
| **Prediction of key allosteric functional residues of enzymes** | | | |
| Confusion matrix | | Predict | |
|  |  | Positive | Negative |
| Real | Positive^a^ | 22 | 51 |
|  | Negative^b^ | 40 | 229 |
| ^a^Positive: The known real positive data is very limited. ^b^Negative: There is little known real negative data, and we regarded data with unknown functions as negative data. | | | |
