# Supplementary material for: Coevolution-based prediction of key allosteric residues for protein function regulation
Source: eLife. 2023 Feb 17;12:e81850. doi: 10.7554/eLife.81850 (PMC9981151; doi:10.7554/eLife.81850)
Supplement: Supplementary file 9. [file elife-81850-supp9.docx]

**Supplementary File 9-** **Comparison of KeyAlloSite and SCA methods**

**Supplementary File 9**. Comparison of KeyAlloSite and SCA methods

| Proteins | Known key allo-residues | SCA^a^ | KeyAlloSite^b^ |
| --- | --- | --- | --- |
| BCR-ABL1 | L359 | N | L359 |
| Tar | Y149, Q152 | Y149, Q152 | Y149, Q152 |
| PDZ3 | A347, L353 | A347, L353 | A347, L353 |
| AurA | T288, T287, S342 | N | T288, T287 |
| CALB | A225^c^ | N (32.8%)^c^ | A225 (38.5%)^c^ |
| CMs | L40, L41, R44, D50, D83, L92, Q93, H95 | D83 | R44, L40 |
| ^a^SCA: Known key allo-residues contained in sectors predicted by SCA. "N" indicates that sectors do not contain known allo-key residues. ^b^KeyAlloSite: The known key allo-residues predicted by KeyAlloSite. ^c^A225: Since CALB contains many key allo-residues, only a typical key allo-residue A225 that has a great impact on enzyme activity is listed here. "N" means that A225 is not included in sectors, and the numbers in brackets represent the proportion of known key allo-residues predicted by SCA and KeyAlloSite. | | | |
